# Grand Sequences

**Published:** 1876-08

**Authors:** James McCarroll


### *GRAND SEQUENCES.*

BY JAMES McCARROLL.

In the discovery of the art of printing, we find to have been laid the first true corner-stone of modern civilization and refinement. At the commencement of the fifteenth century, all Europe was steeped to the lips in ignorance and superstition; and it was not until Guttenberg and Faust, or Fust, had given us moveable types and set up a press at Mentz, that even the feeblest ray of light began to illumine the masses. These types were made of wood, and were first brought to bear upon the printing of the Latin Bible.

They were necessarily clumsy and imperfect; but they served to fire the train whose lustre bathes two hemispheres in intellectual glory to-day.

It is supposed that the first type used was intended to imitate writing, and that it, consequently, partook of the character of script and gothic. It would appear, however, that it met all the necessities of the period; for, as yet there was nothing approaching correct orthography or punctuation. The language was not divided into paragraphs. Capital letters were not used to commence a sentence or to begin proper names. But one kind of letter was used throughout, with a space left at the beginning of chapters for the illuminator, who wrote the initial letter in inks of various colors. Pages had neither running title nor number; and the division of words and sentences was most imperfect. The first presses used were made on the plan of the common wine press. For a brief period, the paper was printed on one side only, the blank sides being subsequently pasted together. Folio and quarto, were the only form of books. Dates were often omitted; and the printer's name appeared at the end of the work, instead of on the title page; while an edition of a couple of hundred copies was considered quite large. Then, there was but few printed books in the world within the reach of a poor man; nor was there, in Europe, a single manuscript that he could read or call his own.

D'Israeli, the elder, who was fond of difficult calculations, informs us that, from the year fourteen hundred and sixty, or something like that date, down to the year eighteen hundred and sixteen, the number of volumes issued by the presses of Europe amounted to at least 3,277,640,000. This was, of course, an immense stride forward: but how utterly disproportioned is it, when compared with the progress made in this direction even in the last fifty years of our own times. For, perhaps, it would not be extravagant to assert that owing to the rapid multiplication of hand and steam presses throughout all civilized lands, and the perfection and cheapness which characterize the founding and the purchase of type of every discription, a greater number of copies of printed books and of other publications have been issued by the press during this comparatively brief period than had ever appeared previously in connection with the annals of the human race. Only fancy, at the time of Guttenberg, not more than a hundred impressions from the press in an hour, and not more, perhaps, than sixty-four letters of the smallest type to the square inch; while in the present day, some of our power presses throw off eleven or twelve thousand impressions in a single hour, with one thousand four hundred and eleven letters to the square inch, should we desire it. And hence the possible correctness of the proposition in relation to the work done within the last half century; or, at most, since November the 28th, 1814, when, in London, England, a newspaper was, for the first time in the history of the art, printed by machinery driven by steam power.

The coincidence is not a little curious, that when the printing presses of Europe had become so improved as to enable them to supply the intellectual needs of all the peoples of the Old World, the locomotive or railway engine, then but recently invented, had just begun to attain that degree of speed and carrying power which soon rendered it a most efficient agent in the scattering, with rapidity, and precision, not only all sorts of printed matter, but articles of trade and commerce from shore to shore. Were it not for the opportune appearance of this swift and sure-footed messenger, at this very moment, so to speak, when the facilities of the press, for the production of books, newspapers, and periodicals, were increased a thousand fold, the diffusion of knowledge would have been greatly retarded, inasmuch as the snail's pace and the restricted capacity of the *diligence*, or the old stage coach could never have met the necessities of the case, or have secured anything like rapid widespread circulation or extensive sales. The printing press, notwithstanding may be regarded as one of the grand corner stones of modern civilization ; while it can be asserted, with equal truth, that it is indebted to this America of ours for the perfection which distinguishes it in the present day and generation.

From the fact that our intellectual needs have been always sacrificed to our physical, the improvements made in the printing press during the space of four hundred years, were significant compared with those made in the locomotive within our memory. True, that the railway engine invented in 1802, by Trevethick, and whose greatest speed was but five miles an hour, was comparatively little in advance of the wooden press of Guttenberg ; but then, while it took centuries to bring the latter to its present state of perfection, the former became almost all that could be desired within the space of sixty or seventy years; and simply because it affected more immediately our material interests. To what extent soever we may starve the soul, the body must be looked after pretty sharply; and hence the amount of time and labor, and thought so generously and freely expended by us upon any project or device calculated to minister more directly to our mere physical comforts or interests.

It would be interesting to note the progress of the railway, from the time when, in 1630, Mr. Beaumont laid down wooden rails from his coal-pits, near Newcastle, to the river-side, in England, to the period when the Pacific or the New York Central was built on this side of the Atlantic. And here it strikes us there is some similarity between certain points in the history of the railway, and that of printing. For in the one case, we first had wooden rails, then metal ones, together with a rudimentary locomotive, which was in due course succeeded by the matchless iron horse of the present time. In the other, we had wooden types which ultimately gave place to metal ones; and had a very primitive press that, in the lapse of many generations, was merged in that triumph of human ingenuity that now performs a miracle at every beat of our pulse. And the similitude seems to hold good for some little distance farther; inasmuch, as the slow rate at which the old hand press threw off a printed sheet, answered to the funereal pail of George Stephenson's Killingworth engine, which was but four miles an hour; while, on the other hand, the superb machine that now produces eleven or twelve thousand printed impressions in sixty minutes, seems to vie in speed with the great iron Cyclops that comes down through the night, at the fearful speed of seventy-five miles an hour. Be this as it may, the railway locomotive, may also be regarded as one of the great corner stones of our civilization, from the fact, that its tendency is to knit nations that are contiguous to each other into one common brotherhood, and to effect between them that speedy exchange of commodities, and interchange of thought which so tend to establish peace on earth and good will toward men.

In the further pursuit of this train of sequences, we shall find upon deliberation, that when the locomotive had attained something like the climax of its mission on land, and brought the various peoples scattered over vast areas into friendly and close contact with each other, the ocean steamer appeared on the scene, and taking up the end of the chain that was dropped by the iron horse on the shores of the sea, stood out with it, against wind and tide, toward the setting sun. From this latter hour, the ends of the earth have been so drawn together that the dusky child of farther India or of the Flowery Land is no longer a stranger at our board, while in any of the great centres of population, all the products of the world find a common mart and are treated as though the term, exotic were no longer of any significance in our language. Through the instrumentality of this mighty agent," the blue gulf of the sea has been shriveled to a span" and lands that were once widely apart, brought almost within hailing distance of each other. Of a truth, within the last twenty-five years, all the nations have struck hands in the cause of human progress and the unity of the race,—a sublime exemplification of which, we shall, during this very year of grace, witness upon these favored shores of ours. At the present day so interwoven is land and water, through the great agencies now under consideration, that a network of civilization may be said to envelop the whole globe. So that ere this the nineteenth century shall have closed, the various peoples of the earth shall stand shoulder to shoulder and learn of each other on a common platform, as it were, until, at last, the angular asperities of creed and caste, shall, through constant and friendly attrition, be so worn away that the east and the west, the north and the south shall be merged in one universal embrace. That like the printing press and the locomotive the ocean steamship shall have played a prominent part in this grand scheme of progress, peace and unity, none will deny ; so that we may, without the slightest hesitation, set it down a third corner stone of the grand fabric which seems so speedily hastening toward completion.

One more corner stone and we shall bring this brief article to a close, and that is to be found in the electric telegraph, which, when the speed of the locomotive and that of the ocean steamer was found not to meet all the necessities of the restless brain and aspirations of the age, stepped in and annihilated time and space. This sub lime triumph of mind over matter is, seemingly the climax of all human ingenuity, with regard to the interchange of thought between the various inhabitants of the earth. Through its mysterious agency all civilized people are brought together, so to speak, in the twinkling of an eye, and shall soon compare notes from St. Petersburgh to Hobart Town ; from the Gulf of Guinea to Borneo, within the self same hour. The gigantic importance of this fact and its bearing upon the future welfare of the whole human family, is absolutely overwhelming. While comtemplating it, we are lost in wonder and amazement, and are totally unable to speculate farther as to what may succeed this last corner stone of our civilization.

